# Efficient Arrangement of the Replication Fork Trap for In Vitro Propagation of Monomeric Circular DNA in the Chromosome-Replication Cycle Reaction

**DOI:** 10.3390/life8040043

**Published:** 2018-09-25

**Authors:** Tomonori Hasebe, Kouhei Narita, Shiomi Hidaka, Masayuki Su’etsugu

**Affiliations:** Department of Life Science, College of Science, Rikkyo University, 3-34-1 Nishi-Ikebukuro, Toshima-ku, Tokyo 171-8501, Japan; 18LD012D@rikkyo.ac.jp (T.H.); grampus.13.k.n@gmail.com (K.N.); s-hidaka@labo.med.osaka-u.ac.jp (S.H.)

**Keywords:** DNA amplification, chromosome replication, replication fork, replication termination

## Abstract

Propagation of genetic information is a fundamental prerequisite for living cells. We recently developed the replication cycle reaction (RCR), an in vitro reaction for circular DNA propagation, by reconstitution of the replication cycle of the *Escherichia coli* chromosome. In RCR, two replication forks proceed bidirectionally from the replication origin, *oriC*, and meet at a region opposite *oriC*, yielding two copies of circular DNA. Although RCR essentially propagates supercoiled monomers, concatemer byproducts are also produced due to inefficient termination of the replication fork progression. Here, we examined the effect of the Tus*-ter* replication fork trap in RCR. Unexpectedly, when the fork traps were placed opposite *oriC*, mimicking their arrangement on the chromosome, the propagation of circular DNA was inhibited. On the other hand, fork traps flanking *oriC* allowed efficient propagation of circular DNA and repressed concatemer production. These findings suggest that collision of the two convergence forks through the fork trap is detrimental to repetition of the replication cycle. We further demonstrate that this detrimental effect was rescued by the UvrD helicase. These results provide insights into the way in which circular DNA monomers replicate repetitively without generating concatemers.

## 1. Introduction

Self-replication is a fundamental property of living systems inherited from the origin of life. To achieve self-replication, cells must replicate their genetic information. In vitro reconstruction is a powerful approach to understand the self-replication event [1,2]. Although polymerase chain reaction (PCR) is an enabling technology to propagate genetic information in vitro through a repetitive cycle of DNA replication, it requires artificial thermal cycling at high temperatures. On the other hand, living cells can continuously propagate large-sized genomic DNA under isothermal conditions through an enzymatic repetition of replication cycles involving large numbers of proteins.

In *Escherichia coli*, replication of the circular chromosome starts from a single replication origin, *oriC*. Two replication forks proceed bidirectionally from *oriC* and eventually merge in a chromosome terminus region that is located approximately opposite *oriC* on the circular map, thereby generating two copies of the circular chromosome [3,4]. A series of replication reactions have been reconstituted in vitro using purified proteins and a circular DNA containing *oriC* [5,6]. The DnaA protein binds to *oriC* and initiates replication by unwinding the DNA duplex [7,8]. DnaB helicase then expands the unwound region to allow association of the replication fork machinery, including single-stranded DNA-binding protein (SSB), DnaG primase, and DNA polymerase III holoenzyme [9,10], the latter of which synthesizes the leading and lagging strand concurrently. Bidirectional progression of two replication forks from *oriC* has been demonstrated in the reconstituted replication system [11,12,13,14].

To control termination of replication, the *E. coli* chromosome (4.6 Mb) contains ten *ter* (termination) sites within the 2 Mb region opposite *oriC* [15,16,17]. Tus binds to *ter* and blocks replication fork progression in an orientation-dependent manner [18,19,20,21]. The chromosomal orientation of *ter* sites is arranged to allow replication forks from *oriC* to enter, but not exit, the termination region. Although the Tus-*ter* fork trap can restrict forks travelling in the *ter*-to *oriC* direction, it does not appear to be directly involved in the completion of replication because *tus*-deficient cells complete chromosome replication and grow normally [22]. Replication fork arrest by the Tus-*ter* system has been demonstrated in the reconstituted *oriC* replication system using a circular DNA containing *ter* sites opposite *oriC* [11,12,14]; however, the effect of the fork trap on repetition of the replication cycle remains unclear.

We recently developed the replication cycle reaction (RCR) by in vitro reconstitution of the *E. coli* chromosome-replication cycle. RCR consists of replication initiation at *oriC*, bidirectional progression of replication forks, completion of replication, and segregation of two daughter circular DNA molecules [23]. The segregation process produces monomeric circular DNA that is topologically identical to the input template DNA, thus allowing autonomous repetition of the replication cycle under isothermal conditions. RCR can propagate circular DNA exponentially with a doubling time of approximately 8 min, even from a single DNA molecule. Because RCR uses the chromosome-replication system, large DNAs (longer than 200 kb) can be propagated as intact circular DNA molecules, and the replication fidelity is extremely high (approximately 1.2 × 10^−8^ errors per base per cycle) [23]. Although RCR produces supercoiled monomer molecules, larger-sized concatemer molecules are partially produced due to rolling circle replication caused by inefficient termination of the replication fork progression.

Here, we investigated the use of the Tus-*ter* fork trap to specifically prevent the production of unwanted concatemer DNA molecules in RCR. We found that fork traps positioned opposite *oriC*, mimicking their arrangement on the chromosome map, severely inhibited DNA propagation. By contrast, positioning of fork traps at a region flanking *oriC*, rather than the termination site, allowed DNA propagation without concatemer formation. Furthermore, using UvrD helicase in RCR eliminated the inhibitory effect of the fork traps on the propagation of supercoiled monomers. The results presented here demonstrate a role of the fork trap in the repetitive cycle of circular DNA replication in an in vitro reconstituted system.

## 2. Materials and Methods

### 2.1. Replication Cycle Reaction

**Figure 1:**
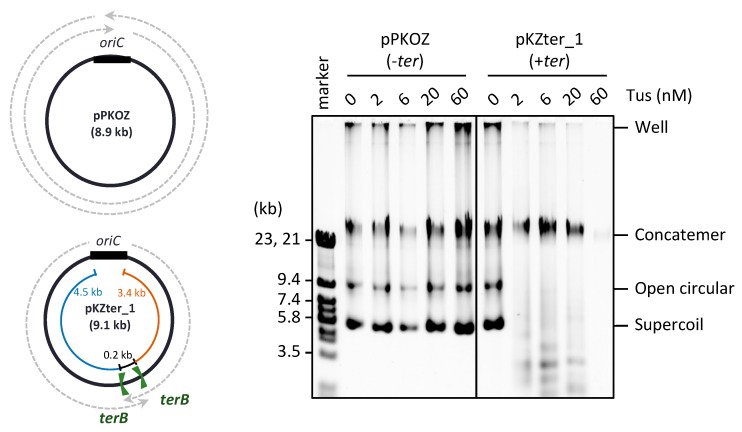
Effect of Tus on the RCR propagation of circular DNA in which the *oriC-ter* arrangement mimics the chromosome position. pKZter_1 (+*ter*) is a pKOZ (*−ter*) derivative containing inward-facing *ter* sites opposite *oriC*. The regions available for fork progression from the *oriC* cassette are indicated by dotted arrows on the circular map. pPKOZ or pKZter_1 (2.5 ng) was incubated in the RCR mixture at 30 °C for 3 h in the presence of the indicated concentrations of Tus. Aliquots (0.2 µL) were analyzed by 0.5% TBE-agarose gel electrophoresis and SYBR Green I staining. The linear DNA size marker fragments were derived from lambda phage DNA (marker).

RCR was performed essentially as described previously [23]. A 10× RCR buffer (200 mM Tris-HCl pH 8.0, 1.6 M potassium acetate, 100 mM Mg(OAc)_2_, 40 mM dithiothreitol (DTT), 40 mM creatine phosphate, 10 mM each NTP, 1 mM each dNTP, 0.5 µg/mL yeast tRNA, 2.5 mM NAD+, 100 mM ammonium sulfate, and 1 mM Tiron) and a 5× Enzyme mix (2.5 mg/mL bovine serum albumin, 100 ng/mL creatine kinase, 0.5 mM ATP, 2 µM SSB_4_, 280 nM IHF_2_, 1 µM DnaG, 200 nM DnaN_2_, 70 nM Pol III*, 100 nM DnaB_6_ C_6_, 500 nM DnaA, 22 nM RNaseH, 140 nM ligase, 170 nM Pol I, 250 nM gyrase (GyrA_2_ B_2_), 25 nM Topo IV (ParC_2_ E_2_), 200 nM Topo III, and 200 nM RecQ) were prepared. The 5 µL reaction mixture was assembled on ice and included 10× RCR buffer (1 µL), 5× Enzyme mix (0.5 µL), and the indicated amount of Tus or Cre. After the addition of *oriC* circular DNA, the reaction was incubated at 30 °C or 33 °C for 3 h, and then stopped by diluting 10-fold with Stop buffer (25 mM Tris-HCl pH 8.0, 25 mM EDTA, 0.1% sodium dodecyl sulfate, 0.05 mg/mL proteinase K, 5% glycerol, and 0.1% bromophenol blue). Preparation lot of 5× Enzyme mix used in Figure 1 was different from that in the other Figures, and the RCR activity was slightly different. An aliquot (2 µL) was analyzed by 0.5% or 1.0% agarose gel electrophoresis, followed by SYBR Green I staining (Molecular Probes, Eugene, USA). The images were acquired with a Typhoon FLA 9500 scanner (GE Healthcare, Uppsala Sweden). The band intensity was quantified to calculate the ratio of concatemers to the sum of concatemers and supercoils. All experiments were performed at least twice, and a representative example is shown.

### 2.2. Purified Proteins

UvrD (82 kDa) was overproduced in BL21 (DE3) cells harboring pET-UvrD, which was constructed by cloning the *uvrD* gene into the pET vector. The cells were grown to OD_600_ = 0.75 in LB medium containing 50 µg/mL carbenicillin, and then incubated for a further 2 h with 0.4 mM IPTG. The cells were then lysed in Lysis buffer (50 mM Tris-HCl pH 8.0, 10% sucrose, 480 mM NaCl, 10 mM spermidine, 2 mM DTT, 20 mM EDTA, 0.25 mg/mL lysozyme, and 100 mM PMSF). After removal of nucleic acids by precipitation with 0.25% polyethyleneimine, ammonium sulfate (0.16 g/mL) was added to the lysate supernatant to precipitate the protein. The ammonium sulfate precipitate was resuspended in Column buffer (50 mM Tris-HCl pH 7.5, 20% glycerol, 5 mM DTT, and 1 mM EDTA) and applied to a HiTrap Heparin column (GE Healthcare) equilibrated with Column buffer containing 100 mM NaCl. After a wash step, the UvrD fractions were eluted with a gradient of 100–500 mM NaCl in Column buffer and pooled. Tus and other replication proteins were purified as described previously [23]. Cre recombinase was purchased from New England Biolabs (MA, USA).

### 2.3. Plasmid Construction

The pPKOZ plasmid (8.9 kb), which contains a 1 kb *oriC* cassette, a *lacZ* gene, and a kanamycin resistance gene, was constructed as described previously [23]. For construction of pPKOZ derivatives (pKZter_1–6, pKZter_5′ter, and pKZter_3′ter), a PKOZ fragment (8.9 kb) was PCR-amplified using pPKOZ as a template and primers SUE1254/SUE1255 targeting a region spanning 4.5 kb upstream and 3.4 kb downstream of the *oriC* cassette. A series of *ter*-cassettes, each containing *terB* sequences (5′-AGTATGTTGTAACTAAAG-3′) and 20 bp overlapping ends for subsequent seamless ligation with the ends of the PKOZ fragment through an originally developed method (Recombination Assembly; to be published elsewhere), were PCR-amplified from pOriDif (12.3 kb) [23] with *terB*-containing primers. For pKZter_1 (9.1 kb), *ter*-cassette_1 (0.2 kb) was amplified with primers SUE1256_1/SUE1257_1. For pKZter_2 (9.4 kb), *ter*-cassette_2 (0.5 kb) was amplified with primers SUE1256_2/SUE1257_2. For pKZter_3 (9.9 kb), *ter*-cassette_3 (1 kb) was amplified with primers SUE1256_3/SUE1257_3. For pKZter_4 (10.9 kb), *ter*-cassette_4 (2 kb) was amplified with primers SUE1256_4/SUE1257_4. For pKZter_5 (12.9 kb), *ter*-cassette_5 (4 kb) was amplified with primers SUE1256_5/SUE1257_5. For pKZter_6 (19.0 kb), *ter*-cassette_6 (10 kb) was amplified with primers SUE1322/SUE1323. For pKZ_5′ter (9.1 kb), *ter*-cassette_5′ter (0.2 kb) was amplified with primers SUE1509/SUE1257_1. For pKZ_3′ter (9.1 kb), *ter*-cassette_3′ter (0.2 kb) was amplified with primers SUE1256_1/SUE1510. The ligation products were subjected to transformation of *E. coli* strain DH5α, and each *oriC* plasmid was recovered from a kanamycin-resistant colony.

For construction of pCLter30k, the Lter11 (a 14.8 kb chromosomal region from *argS* to *folE*) and Lter17 (a 14.8 kb chromosomal region from *yeaI* to *dmlA*) fragments were PCR-amplified from *E. coli* strain Δ33a [24] with primers Lter1f/Lter5r_2OL and SUE972/SUE973, respectively. The OL cassette (451 bp), which contained *oriC* sequences (286 bp) and *loxP* sequences (5′-ATAACTTCGTATAGCATACATTATACGAAGTTAT-3′), was prepared through polymerase cycling assembly [25] using oligo DNAs AO15, AO16-loxP, AO17, AO18, AO19, and AO20, with amplification primers LterOf/LterOr. To generate the Cm-OL fragment (1.2 kb), a chloramphenicol-resistant (Cm) cassette (0.75 kb) was PCR-amplified from pACYC184 with primers LterCf_2/LterCr_2, and then ligated with the OL cassette through overlap PCR with amplification primers LterCf_2/SUE986. The LterCf_2 and SUE986 primers contained 60 bp overlapping ends for subsequent seamless ligation with the ends of the Lter11 and Lter17 fragments, respectively. The *ter*-cassette_L (0.2 kb) was PCR-amplified from pOriDif with primers SUE1700 and SUE1559, which contained the *terB* sequences and 40 bp overlapping ends for subsequent seamless ligation with the ends of the Lter11 and Lter17 fragments, respectively. The four fragments (Lter11, Cm-OL, Lter17, and *ter*-cassette_L) were simultaneously assembled through Recombination Assembly. The assembly products were subjected to transformation of *E. coli* strain DH5α, and the pCLter30k plasmid (30.9 kb) was recovered from a chloramphenicol-resistant colony.

For construction of pCLter_1 and pCLter_2, the OLDT cassette (424 bp) was constructed by PCR amplification of the OL cassette with *terB*-containing primers SUE1046/SUE1047. For the Cm-OLDT fragment (1.2 kb), the Cm cassette (0.75 kb) was ligated with the OLDT cassette through overlap PCR with primers LterCf_2/SUE1047. The DCW2 fragment (1.1 kb) and DCW6 fragment (3.2 kb), which consisted of a part of a region on the *E. coli* chromosome called the division and cell wall (*dcw*) cluster [26], were PCR-amplified from the MG1655 genome with primers SUE1158/SUE1161 and SUE1158/SUE1110, respectively. Subsequently, 60 bp overlapping sequences were added to the Cm-OLDT fragment for seamless ligation with the DCW2 or DCW6 fragment, through PCR amplification with primers SUE1096/SUE1313 or SUE1096/SUE1314, respectively. The ligation products were subjected to transformation of *E. coli* strain DH5α, and pCLter_2 (a 2.3 kb plasmid consisting of Cm-OLDT and DCW2) and pCLter_1 (a 4.5 kb plasmid consisting of Cm-OLDT and DCW6) were recovered from chloramphenicol-resistant colonies.

For construction of pUC_OLDT and pUC_OriC300, the pUC fragment (2.7 kb) was PCR-amplified from pUC19 with primers SUE1156/SUE1361, which annealed to opposite sequences located immediately downstream of the *lacZ*α gene. The OLDT2 cassette (544 bp) and *oriC*300 control cassette (481 bp), each containing 40 bp overlapping ends for subsequent seamless ligation with the pUC fragment, were PCR-amplified with primers SUE1684/SUE1685 from the OLDT cassette or the 1 kb *oriC* cassette, respectively. The ligation products were subjected to transformation of *E. coli* strain DH5α, and pUC_OLDT (a 3.1 kb plasmid consisting of OLDT2 and pUC fragments) and pUC_OriC300 (a 3.0 kb plasmid consisting of *oriC*300 and pUC fragments) were recovered from ampicillin-resistant colonies.

The primer sequences are listed in Supplementary Table S1. All plasmids were purified using the QIAprep Miniprep Kit (Qiagen, Venlo, The Netherlands), and the *ter* sequences were verified by DNA sequencing.

## 3. Results

### 3.1. Chromosome-Like Arrangement of oriC-ter Sites Is Detrimental to the RCR Propagation of Circular DNA

In our previous studies of RCR, we found that propagation of supercoiled monomers of circular DNA is inhibited slightly by Tus-*ter* fork traps positioned opposite *oriC*, while the fork traps efficiently block the generation of concatemer byproducts due to rolling circle replication [23]. In RCR, the circular DNA has two inverted *ter* sites with the non-permissive sides facing each other (inward-facing), such that the arriving fork originating from *oriC* is permitted to pass through, but the fork leaving the termination region is blocked, thereby mimicking the *oriC-ter* arrangement on circular chromosomes. In the previous experiment, RCR was performed at 30 °C for only 1 h, during which the replication cycle repeats up to approximately seven times, as deduced from the doubling time of circular DNA (approximately 8 min) [23]. When the incubation time was extended to 3 h, inhibition of the propagation of target supercoiled monomers containing inward-facing *ter* sites (pKZter_1) became much more severe (Figure 1). In reactions using circular DNA lacking *ter* sites (pPKOZ), RCR efficiently propagated concatemers and supercoiled monomers, and no apparent inhibition by Tus was detected. These observations indicate that inward-facing *ter* sites positioned opposite *oriC* are detrimental to repetition of the replication cycle in RCR.

### 3.2. The Arrangement of ter Sites, Rather Than the Distance between Them, Is Critical for Repetition of the Replication Cycle

**Figure 2:**
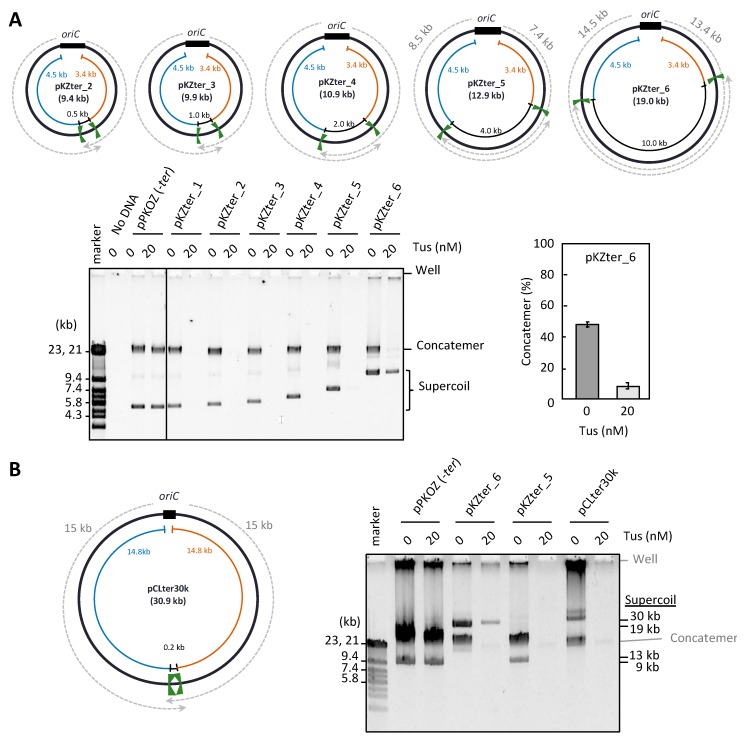
Effect of the inter*-ter* length on the RCR propagation in the presence of Tus. (**A**) pKZter_2–6 are pKZter_1 derivatives in which the length between the inward-facing *ter* sites was extended to 0.5, 1.0, 2.0, 4.0, or 10 kb. The circular maps are shown as in Figure 1. The indicated plasmid (2.5 ng) was incubated in the RCR mixture at 30 °C for 3 h in the absence (0 nM) or presence (20 nM) of Tus. The product was analyzed by 0.5% TBE-agarose gel electrophoresis and SYBR Green I staining. The ratio of concatemers to the sum of concatemers and supercoils is shown in a graph as the average value of three repetitive experiments with standard deviation. (**B**) In pCLter30k, the regions available for fork progression were 15 kb (dotted arrows), while the inter-*ter* length was 0.2 kb. RCR was performed as in (**A**), except that a 1% agarose gel was used to separate large supercoiled DNAs from concatemers. Large supercoiled DNA migrates more slowly than linear DNA [29]. DNA size marker fragments were derived from lambda phage DNA (marker).

The minimum distance between the two inward-facing *ter* sites on the *E. coli* 4.6 Mb chromosome is 267 kb (the distance between *terA* and *terC*) [15]. On the other hand, the distance between the *ter* sites in the circular DNA we tested (pKZter_1) was much shorter (0.2 kb). Therefore, we examined whether a certain inter-*ter* distance is required for continuous repetition of the replication cycle in RCR. Five additional circular DNAs (pKZter_2–6), in which the distance between the two inward-facing *ter* sites was extended to 0.5, 1, 2, 4, or 10 kb, were constructed by inserting various lengths of DNA into the inter-*ter* region. Propagation of the circular DNA was still inhibited by Tus when the inter-*ter* distance was extended to 4 kb (pKZter_5), but a 10 kb inter-*ter* distance (pKZter_6) allowed efficient propagation of supercoiled monomers, even in the presence of Tus (Figure 2A). Furthermore, Tus severely repressed the production of unwanted concatemers while allowing propagation of the supercoiled monomers of pKZter_6, indicating that the Tus-*ter* traps operated properly to repress rolling circle replication.

There are at least two possible explanations for how pKZter_6 was able to escape the inhibitory effect of Tus-*ter* traps on repetition of the replication cycle. The first is that the longer distance between *oriC* and the trap position for each bidirectional fork (14.5 and 13.4 kb for pKZter_6, in contrast to 8.5 and 7.4 kb for pKZter_5; see plasmid maps in Figure 2A) provides more time for the second fork to start from *oriC* before the first fork reaches the trap site. Unidirectional fork progression can take place in the early stage of replication in the in vitro *oriC* replication reaction [27]. Because the fork speed is approximately 0.5 kb/s [28], the fork that started earlier would reach the trap site in pKZter_5 in approximately 15 s (based on a distance of 7.4 kb). In this case, the situation in which one arm of the fork is trapped before the other arm leaves *oriC* may result in detrimental effects on replication of the replication cycle. To test this hypothesis, we constructed pCLter30k, a long arm version of pKZter_1 that had a 15 kb region between *oriC* and the non-permissively oriented *ter* site for each left and right arm, while the distance between the two inward-facing *ter* sites remained unchanged (0.2 kb). Unexpectedly, extension of the left and right arm distances did not overcome the inhibitory effect of Tus on RCR propagation (Figure 2B).

**Figure 3:**
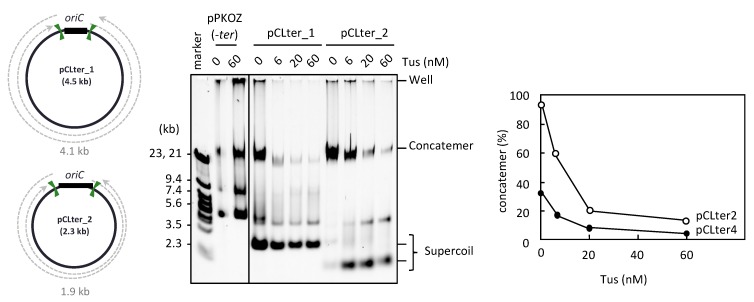
RCR propagation of circular DNA containing *ter* sites on both sides of *oriC*. The circular maps of pCLter_1 and pCLter_2 are shown as in Figure 1. The indicated plasmid (2.5 ng) was incubated in the RCR mixture at 30 °C for 3 h in the absence (0 nM) or presence (6, 20, or 60 nM) of Tus. The product was analyzed by 0.5% TBE-agarose gel electrophoresis and SYBR Green I staining. The ratio of concatemers to the sum of concatemers and supercoils is shown in a graph.

The second possible explanation for how pKZter_6 was able to escape the inhibitory effect of Tus-*ter* traps on repetition of the replication cycle involves the arrangement of the two *ter* sites on the pKZter_6 circular map, in which each *ter* site is in close proximity to *oriC* rather than the site opposite *oriC*. The inter-*ter* distance thus occupies more than 50% of total plasmid size, in contrast to that of pKZter_5 (31%). Such an arrangement may increase opportunities for the two bidirectional forks to meet and merge each other before being trapped at the *ter* site, while an unwanted portion of forks that escape from the fork merge event are trapped, thereby repressing the rolling circle replication. This second explanation was addressed using pCLter_1 and pCLter_2. These DNA constructs contained two *ter* sites flanking both sides of *oriC*, with an orientation permitting the forks to leave but not enter *oriC*. In addition, the distances between the two inward-facing *ter* sites in pCLter_1 and pCLter_2 (4.1 kb and 1.9 kb, respectively) were as short as those in pKZter_5 and pKZter_4, respectively, the propagation of which was inhibited by Tus (see above). As shown in Figure 3, pCLter_1 and pCLter_2 were propagated as supercoiled monomers and concatemer production was suppressed in the presence of Tus, supporting the idea that the inhibition of supercoiled monomer propagation by Tus in the previous experiments was caused by fork merging through the Tus-*ter* trap. We also observed that, for the smaller-sized DNA, pCLter_2 (2.3 kb), production of the supercoiled monomers was inefficient in the absence of Tus, and rolling circle replication (i.e., concatemer production) was predominant. Propagation of the supercoiled monomers of pCLter_2 was enhanced by Tus, probably due to the fact that the repression of rolling circle replication can increase the amount of replication components available for the replication cycle mode of circular DNA.

**Figure 4:**
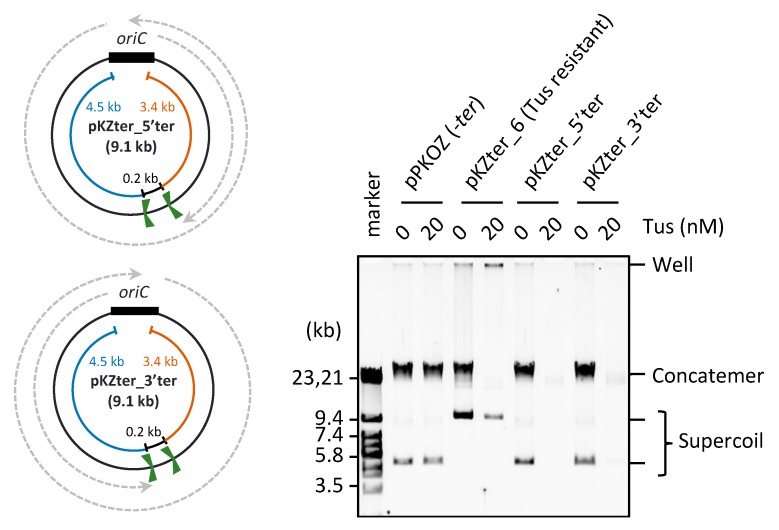
Effect of two same direction *ter* sites placed opposite *oriC*. The circular maps of pKZter_5′ter and pKZter_3′ter are shown as in Figure 1. The indicated plasmid (2.5 ng) was incubated in the RCR mixture at 30 °C for 3 h in the absence (0 nM) or presence (20 nM) of Tus. The product was analyzed by 0.5% TBE-agarose gel electrophoresis and SYBR Green I staining.

Considering that fork merging through the Tus-*ter* trap is detrimental in RCR, we hypothesized that the detrimental effect would be detected even when the two *ter* sites positioned opposite *oriC* are oriented in the same direction. To test this possibility, we constructed pKZter_5’ter and pKZter_3’ter, pKZter_1 derivatives with two right- or left-oriented *ter* sites positioned opposite *oriC* and separated by 0.2 kb (Figure 4). As expected, in the presence of Tus, RCR propagation of pKZter_5′ter and pKZter_3′ter was inhibited, whereas the control pKZter_6 DNA was propagated as supercoiled monomers. When the contrast of the original image was increased, slight propagation products were detected in the reactions using pKZter_5′ter or pKZter_3′ter, but not in the reaction using pKZter_1-5 (Supplementary Figure S1). This result is reasonable considering that the chance of fork merging without mediating the Tus-*ter* trap could increase in the situation where the traps halt only a fork coming from one direction, compared with the situation where forks from both directions are halted.

### 3.3. UvrD Helicase Rescues the Detrimental Effect of the Tus-ter Traps in RCR

**Figure 5:**
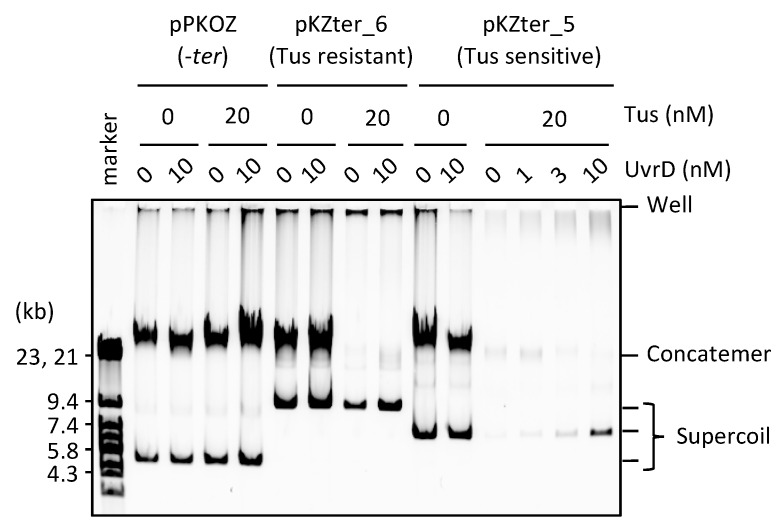
UvrD helicase rescues the detrimental effect of Tus in RCR. pPKOZ (*−ter*), pKZter_6 (Tus resistant), or pKZter_5 (Tus sensitive) was subjected to RCR propagation in the absence or presence of Tus as described in Figure 2A, except that the indicated concentration of UvrD was present in the reaction.

A previous study showed that UvrD helicase is required for growth of cells whose left and right chromosome arms have ectopic *ter* sites in non-permissive orientation for the replication forks progressed from *oriC*, suggesting that UvrD may function in the removal of Tus from *ter* [29]. Therefore, we examined whether UvrD is able to rescue the detrimental effect of Tus on circular DNA propagation in RCR. We used pKZter_5, the propagation of which was inhibited by Tus due to two *ter* sites positioned closer to a region opposite *oriC* than to *oriC* (see above). UvrD enabled pKZter_5 to propagate as supercoiled monomers without producing concatemers, even in the presence of Tus (Figure 5). In a control reaction using pKZter_6, which propagated as supercoiled monomers in the presence of Tus, no marked effect of UvrD was observed. These results suggest that UvrD plays a role in elimination of the detrimental effect of Tus and allows the trapped fork to merge properly for the subsequent processes of the replication cycle.

### 3.4. Resolution of the DNA Concatemers by the Cre-loxP System in RCR

**Figure 6:**
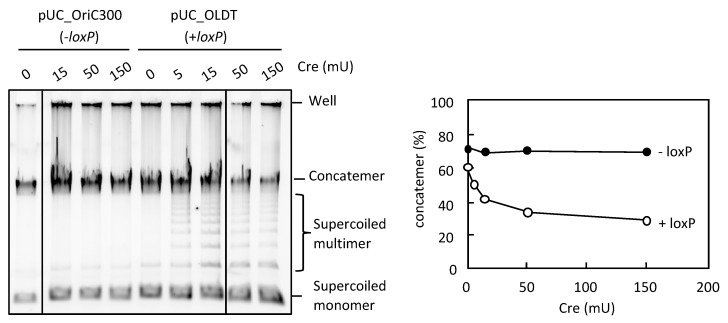
Effect of Cre-*loxP* system on the concatemer production in RCR. pUC_OriC300 (−*loxP*) or pUC_OLDT (+*loxP*) (0.05 ng) was incubated in the RCR mixture at 33 °C for 3 h in the absence (0 mU) or presence (5, 15, 50, or 150 mU) of Cre recombinase. The product was analyzed by 0.5% TBE-agarose gel electrophoresis and SYBR Green I staining. The ratio of concatemers to the sum of concatemers and supercoils is shown in a graph.

Next, we examined an alternative way to reduce concatemer production and enhance supercoiled DNA production in RCR. The Cre/*loxP* site-directed recombination system is an ideal system to resolve concatemer and generate circular DNAs through Cre-mediated recombination between directly repeating loxP sites [30]. We constructed pUC_OLDT (3 kb), which had a *loxP* site flanking *oriC*. When Cre recombinase was present in RCR, a ladder of bands representing supercoiled multimers was detected between the bands of concatemers and supercoiled monomers, along with a partial reduction of the concatemer products (pUC_OLDT; Figure 6). By contrast, there was no marked effect of Cre recombinase when the template circular DNA lacked the *loxP* site (pUC_OriC300; Figure 6). This result indicates that the Cre/*loxP* system can act in RCR to resolve concatemers that are generated due to defective termination of replication fork progression.

## 4. Discussion

In *E. coli*, chromosome replication proceeds bidirectionally from *oriC* and terminates at a region opposite *oriC* on the circular chromosome map through merging of the two converging replication forks. Multiple *ter* sites are located throughout the terminus half of the chromosome [15,16,17]. In this study, we found that Tus*-ter* fork traps located around the termination region are detrimental to repetition of the replication cycle of the *oriC* circular DNA in our in vitro reconstitution system, RCR. When the fork trap was repositioned to a region closer to *oriC*, allowing the forks on the left and right arms to pass through the terminus half of the circular map without being trapped by the Tus*-ter* system, supercoiled monomers were efficiently propagated and the production of unwanted concatemers was suppressed. These findings indicate that the fork merge termination event must occur without mediation of the Tus*-ter* system, in order to repeat the replication cycle in vitro, whereas the Tus*-ter* system acts to efficiently repress rolling circle-type over-replication. Our findings support the previous view that the replication termination can occur in the absence of the fork trap, and the fork trap serves as a safeguard system to halt the over-progression of the replication fork which might be disadvantageous because of the terminus-to-origin direction of replication [31]. In contrast to the effective position of the fork traps demonstrated in this in vitro study, the fork traps in *E. coli* and *B. subtilis* position near the termination region of their chromosome [31]. The direction of gene transcription in these chromosomes are majorly oriented in the origin-to-terminus direction [32]. The fork trap position of the chromosomes would be advantageous in the situation where replication and transcription occur simultaneously for avoiding head-on collision between the replication fork and the transcription machinery. Also, the cellular segregation process, in which several proteins, such as FtsK, Topoisomerase IV, and XerCD are involved, occurs via the *dif* site that is positioned at the terminus [31]. The chromosome arrangement of the fork trap can narrow the region for termination to coordinate with segregation.

Although intermediates leading to the concatemer formation in RCR is not presently clear, unidirectional fork progression and therefore nascent strand displacement by the fork might partly occur, leading to rolling circle replication. For unidirectional replication of the plasmid R1 in vivo, the rolling circle replication products have been observed, which is repressed in the presence of the Tus*-ter* system [33].

It is reported that the fork approaching the non-permissive face of *ter* is trapped by a mechanism in which Tus forms a stable locked complex with the fork structure [20]. The locked complex might impede the fork merge event or the decatenation event, resulting in inhibition of the subsequent replication cycle process. For example, the trapped fork might keep the locked complex state even upon a collision with a fork coming from the other direction until dissociation of the locked complex by mechanisms in which UvrD helicase is probably involved. Alternatively, the trapped fork might easily cause a double-strand break due to collision of the fork coming behind during repetition of the replication cycle. The resultant linear DNA is inactive for replication initiation in RCR [23].

In contrast to the in vitro observations, Tus-sensitive plasmids containing *ter* sites near the termination region (pKZter_1–5, pKZter_5′ter, and pKZter_3′ter) are replicated from *oriC* properly in vivo. These plasmids were stably inherited as *oriC* plasmids in *E. coli* DH5α (*tus*+) and were purified as well as the *oriC* DNAs lacking *ter* sites (data not shown). This observation implies that some mechanisms exist in vivo to overcome the detrimental effect of the fork trap. Indeed, we demonstrated in vitro that UvrD helicase rescues the inhibitory effect of the fork trap and allows repetition of the replication cycle in RCR (Figure 5). Consistent with this finding, UvrD is required for the growth of cells in vivo where replication forks are arrested at ectopic *ter* sites [29]. Whereas the 5′ to 3′ helicase activity of DnaB is halted by the Tus*-ter* trap, it has been demonstrated in vitro that the 3′ to 5′ helicase activity of UvrD is unaffected by this trap [34]. UvrD can initiate DNA unwinding from nicks in vitro [35]. It seems plausible that UvrD is loaded onto a leading strand gap when DnaB on the lagging strand of the fork encounters the fork trap, dissociating the Tus*-ter* locked complex, and thereby allowing the converging forks to merge properly.

When the size of the template circular DNA was relatively small (2.3 kb, pCLter_2), RCR propagation of the supercoiled monomers was inefficient, and concatemer production became predominant (Figure 3). Presumably, in the case of smaller circular DNAs, the fork merge event would occur less frequently because the fork that started earlier can complete replication of such a short template unidirectionally, without waiting for the other fork to start, resulting in rolling circle replication. We also observed that, when rolling circle replication was repressed by the Tus*-ter* trap, supercoiled monomer propagation of pCLter_2 became predominant.

In summary, we have established an efficient way to repress the unwanted concatemer byproducts in RCR by placing Tus*-ter* traps on either side of *oriC*, thereby enabling the supercoiled monomers to propagate predominantly and continuously. Propagation of DNA as individually separated molecules is a crucial event for self-replication of all living organisms. Our RCR system provides a powerful in vitro tool for reconstruction of self-replicating living systems.

## Supplementary Materials

The following are available online at http://www.mdpi.com/2075-1729/8/4/43/s1, Table S1: Primer list. Figure S1: High-contrast image of Figure 2A and Figure 4.
