# Supplementary material for: Efficient Arrangement of the Replication Fork Trap for In Vitro Propagation of Monomeric Circular DNA in the Chromosome-Replication Cycle Reaction
Source: Life (Basel). 2018 Sep 25;8(4):43. doi: 10.3390/life8040043 (PMC6315707; doi:10.3390/life8040043)
Supplement: Supplementary file 1 [file life-08-00043-s001.zip › Supplementary files/Supplementary FIgure S1.docx]

**Supplementary Figure S1.** High-contrast image of Figures 2A and 4. (A) Image from Figure 2A. (B) Image from Figure 4.
