# Supplementary material for: Efficient Arrangement of the Replication Fork Trap for In Vitro Propagation of Monomeric Circular DNA in the Chromosome-Replication Cycle Reaction
Source: Life (Basel). 2018 Sep 25;8(4):43. doi: 10.3390/life8040043 (PMC6315707; doi:10.3390/life8040043)
Supplement: Supplementary file 1 [file life-08-00043-s001.zip › Supplementary files/Supplementary Table S1.docx]

Supplementary Table S1. Primer list

| Name | Sequence (5' -> 3') |  |
| --- | --- | --- |
| SUE1254 | ttccttacgcgaaatacgggca | PKOZ_forward |
| SUE1255 | atgacgacaggatagtctgagggt | PKOZ*_*reverse |
| SUE1256_1 | CCCGTATTTCGCGTAAGGAActttagttacaacatactaagccgagaatacggtagtaagtg | *ter*-cassette_1_forward |
| SUE1257_1 | TCAGACTATCCTGTCGTCATctttagttacaacatactgagaggcttaagttcttgaaccct | *ter*-cassette_1_reverse |
| SUE1256_2 | CCCGTATTTCGCGTAAGGAActttagttacaacatactgagattcccggaagtgcagat | *ter*-cassette_2_forward |
| SUE1257_2 | TCAGACTATCCTGTCGTCATctttagttacaacatacttcattcccgcttgatccgt | *ter*-cassette_2_reverse |
| SUE1256_3 | CCCGTATTTCGCGTAAGGAActttagttacaacatactcagtggttgattggcgcaac | *ter*-cassette_3_forward |
| SUE1257_3 | TCAGACTATCCTGTCGTCATctttagttacaacatactagctatggttaatgtttgcttgctg | *ter*-cassette_3_reverse |
| SUE1256_4 | CCCGTATTTCGCGTAAGGAActttagttacaacatactcagatatcccgctaggcatgat | *ter*-cassette_4_forward |
| SUE1257_4 | TCAGACTATCCTGTCGTCATctttagttacaacatactagctgtaattcagcgaagtcgt | *ter*-cassette_4_reverse |
| SUE1256_5 | CCCGTATTTCGCGTAAGGAActttagttacaacatacttcggtgtgcaactaggtgataaag | *ter*-cassette_5_forward |
| SUE1257_5 | TCAGACTATCCTGTCGTCATctttagttacaacatacttgtgcagccataatcgaggac | *ter*-cassette_5_reverse |
| SUE1322 | CCCGTATTTCGCGTAAGGAActttagttacaacatactgggggaatgtgggaggtc | *ter*-cassette_6_forward |
| SUE1323 | TCAGACTATCCTGTCGTCATctttagttacaacatactgcgcggattttcttacaggt | *ter*-cassette_6_reverse |
| SUE1509 | CCCGTATTTCGCGTAAGGAAagtatgttgtaactaaagaagccgagaatacggtagtaagtg | *ter-cassette_*5'ter_forward |
| SUE1510 | TCAGACTATCCTGTCGTCATagtatgttgtaactaaaggagaggcttaagttcttgaaccct | *ter-cassette_*3'ter_revrse |
| Lter1f | aatagaaaactgccagtgcgca | Lter11_forward |
| Lter5r_2 | ccatcgtcgttggcaaccttttcgc | Lter11_reverse |
| SUE972 | cttaccgaatctgaaacaaaaccaacaacg | Lter17_forward |
| SUE973 | tagctttccattacgccaaagaagaaccag | Lter17_reverse |
| AO15f | ttaatttaaaaggatctaggtgaagatcctttttgataatctcatgaccaaaatcccttaacgtgagttttcgttCCACTGAGAAGATCCGGCAGAAGAA | OL_PCA |
| AO16r-loxP | TCCTGACAGAGTTATCCACAGTAGAtcgcacgatctgtataacttcgtatagcatacattatacgaagttatccaTTCTTCTGCCGGATCTTCTCAGTGG | OL_PCA |
| AO17f | TCTACTGTGGATAACTCTGTCAGGAagcttggatcaaccggtagttatccaaagaacaactgttgttcagtttttGAGTTGTGTATAACCCCTCATTCTG | OL_PCA |
| AO18r | GCTTTTAAGATCAACAACCTGGAAAggatcattaactgtgaatgatcggtgatcctggaccgtataagctgggatCAGAATGAGGGGTTATACACAACTC | OL_PCA |
| AO19f | TTTCCAGGTTGTTGATCTTAAAAGCcggatccttgttatccacagggcagtgcgatcctaataagagatcacaatAGAACAGATCTCTAAATAAATAGAT | OL_PCA |
| AO20r | tccccgaaaagtgccacctgacgtcgtcggcttgagaaagacctgggatcctgggtattaaaaagaAGATCTATTTATTTAGAGATCTGTTCT | OL_PCA |
| LterOf | TACTGCGATGAGTGGCAGGGCGGGGCGTAAgaagatccggcagaagaatgg | OL_forward |
| LterOr | CCGATTTGTCTGTTTAAATTGCATGAACCGGTACAGGTTGCGCACTGGCAGTTTTCTATTgtcggcttgagaaagacctg | OL_reverse |
| SUE1046 | gaagatccggcagaagaatggagtatgttgtaactaaagataacttcgtataatgtatgctatacg | OLTD_forward |
| SUE1047 | gtcggcttgagaaagacctgagtatgttgtaactaaaggtgcgcataatgtatattatgttaaatggatcctgggtattaaaaagaag | OLTD_reverse |
| LterCf_2 | GATTGTACTGGTGATGCCAGACACCAGCCCGCGCGGCGAAAAGGTTGCCAACGACGATGGcgtaagaggttccaactttcacc | Cm_forward |
| LterCr_2 | CCATTCTTCTGCCGGATCTTGttacgccccgccctgccac | Cm_reverse |
| SUE986 | GCCCGATGCGTTTTATATTCGGTCAGCTGGCGTTGTTGGTTTTGTTTCAGATTCGGTAAGgtcggcttgagaaagacctg | Cm-OL_reverse |
| SUE1700 | CCGTCGGTTGCTGGTTCTTCTTTGGCGTAATGGAAAGCTActttagttacaacatactaagccgagaatacggtagtaagtg | ter-cassette_L_foward |
| SUE1559 | GCATGAACCGGTACAGGTTGCGCACTGGCAGTTTTCTATTctttagttacaacatactgagaggcttaagttcttgaaccct | ter-cassette_L_reverse |
| SUE1158 | ttctaaaaccgtgactgcggatatcccgattgtggg | DCW2/6_forward |
| SUE1161 | gaatatttccgtattacgtttcacttcgtcac | DCW2_reverse |
| SUE1110 | ctcgataatttcccattcaggcagggggta | DCW6_reverse |
| SUE1096 | AGGCGGGTGCCCAGCGGGTAGCGGCGGAACTGGGGATTGTACTGGTGATGCCAGACACCAcgtaagaggttccaactttcacc | Cm-OLDT_foward for DCW2/6 |
| SUE1313 | GTGAAAATTCAGGCCAGCGGTTACGGGCGTGACGAAGTGAAACGTAATACGGAAATATTCgtcggcttgagaaagacctg | Cm-OLDT_reverse for DCW2 |
| SUE1314 | ATTGACATTTATCACCCGTGCCTGCTGCTTTACCCCCTGCCTGAATGGGAAATTATCGAGgtcggcttgagaaagacctg | Cm-OLDT_reverse for DCW6 |
| SUE1156 | CTATGCGGCATCAGAGCAG | pUC_forward |
| SUE1361 | GTTAAGCCAGCCCCGACAC | pUC_reverse |
| SUE1684 | ATGGTGCACTCTCAGTACAATCTGCTCTGATGCCGCATAGGaagatccggcagaagaatgg | OLDT2/OriC300_forward |
| SUE1685 | CGCGTCAGCGGGTGTTGGCGGGTGTCGGGGCTGGCTTAACGtcggcttgagaaagacctg | OLDT2/OriC300_reverse |

The *ter* sequences are underlined. Capital letters indicate the overlapping ends.
